# Promoting effect of 1,25(OH)_2_ vitamin D_3_ in osteogenic differentiation from induced pluripotent stem cells to osteocyte-like cells

**DOI:** 10.1098/rsob.140201

**Published:** 2015-02-04

**Authors:** Hiroshi Kato, Hiromi Ochiai-Shino, Shoko Onodera, Akiko Saito, Takahiko Shibahara, Toshifumi Azuma

**Affiliations:** 1 Department of Oral and Maxillofacial Surgery, Tokyo Dental College, Tokyo, Japan; 2 Department of Biochemistry, Tokyo Dental College, Tokyo, Japan

**Keywords:** induced pluripotent stem cells, vitamin D, osteoblasts, osteocytes, drug assessment

## Abstract

We recently reported a new method to purify the induced pluripotent stem (iPS)-derived osteoprogenitors (iPSop). In this paper, we optimized the procedure and characterized cells at each process step. We observed that 10 days of treatment with FGF-2, IGF-1 and TGF-β (FIT) resulted in early-phase osteoblasts and 14 days of treatment resulted in late-phase osteoblasts. We found that treatment with 1,25(OH)_2_ vitamin D_3_ increased expression of osteocalcin and decreased expression of tissue-non-specific alkaline phosphatase and runt-related transcription factor 2 (RUNX2) in iPSop-day14 cells (cells treated with FIT for 14 days). Therefore, iPSop-day14 cells were promoted to mature osteoblasts by 1,25(OH)_2_ vitamin D_3_ treatment. In addition, we found that 1,25(OH)_2_ vitamin D_3_ treatment for 14 days enhanced not only mineralization but also expression of osteocyte markers, including dentin matrix protein-1 and fibroblast growth factor-23, in iPSop cells. Therefore, 1,25(OH)_2_ vitamin D_3_ is a potent promoter of osteoblast–osteocyte transition. The results of this study suggest that it is possible to evaluate both early- and late-phase osteoblasts and to apply cells to drug screening for anabolic drugs that stimulate bone formation.

## 2. Introduction

Induced pluripotent stem cells (iPSCs) can generate a variety of patient-specific cells and are used to explore disease mechanisms and novel therapeutic molecular targets in drug development [1–4]. Recent studies have reported on a number of different types of cells generated from iPSCs [5–7]. We have previously developed an effective procedure to generate not only osteoblastic cells but also osteocyte-like cells [8,9]. Osteocytes, once considered as silent cells trapped in mineralized bone, are now identified as key regulators of bone remodelling [10,11]. Osteocytes are postmitotic, terminally differentiated osteoblasts and many reside in mineralized matrices. The most abundant cell in bone and central to bone remodelling, osteocytes secrete many soluble factors that not only target cells on the bone surface but also target other organs. Osteocytes most abundantly express receptor activator of nuclear factor kappa-B ligand, which functions as a key factor in osteoclast differentiation and activation. Therefore, any drugs targeting osteocyte function and signalling pathways will have a major impact on the bone remodelling process. Currently, much research into drug development, particularly for osteoporosis, is focused on targeting osteocytes. For this reason, the generation of osteocytes has become particularly important.

Osteoporosis is a very common health problem among elderly people and is thought to affect more than 200 million people worldwide [12]. With ageing populations in many countries, the incidence of osteoporosis will increase further. Therefore, it is important to develop novel drugs for the treatment of osteoporosis [13–15]. Osteoporosis is caused by an uncoupling of bone resorption from bone formation and is associated with reductions in bone strength determined by bone mineral density and bone quality. Reductions in bone strength occur because of an imbalance between the activity of osteoclasts and osteoblasts. In addition, bone formation is impaired in elderly people [16–18], and loss of bone mineral density is caused by a decrease in calcium absorption with ageing [19]. Consequently, the microarchitecture of trabecular bone deteriorates [20].

Osteoporosis drugs are categorized into two classes: antiresorptive drugs that inhibit bone resorption and anabolic drugs that stimulate bone formation. The bisphosphonates (BPs) are antiresorptive drugs and are most commonly prescribed for the treatment of osteoporosis [15,16,21,22]. However, BPs may cause bisphosphonate-related osteonecrosis of the jaw, which is a rare but well recognized serious side effect of long-term bisphosphonate use [23–27]. Therefore, there is a need for anabolic drugs that could effectively modulate osteoblast or osteocyte activity to regenerate bone. iPSC technology can be applied to drug assessment and to patient-specific approaches to examine individual differences in pharmacokinetic and pharmacodynamic features.

All therapeutic management strategies for the prevention and treatment of osteoporosis include recommendations for calcium and vitamin D supplementation [28]. The active form of vitamin D, 1,25(OH)_2_ vitamin D_3_, contributes to a wide range of biological activities by binding to the nuclear vitamin D receptor. It is well established that the appropriate use of vitamin D can prevent the progression of osteoporosis. Many studies have shown that vitamin D has both anabolic and catabolic roles in bone homeostasis. However, the effects of vitamin D on bone tissue and bone cells have not yet been entirely evaluated. It is important to assess the precise effects of vitamin D on the osteolineage cells, osteoblasts and osteocytes. Here, we show that human osteolineage cells derived from human iPSCs (hiPSCs) can provide an ideal model to analyse the effects of 1,25(OH)_2_ vitamin D_3_ [9,29–33]. Our results suggest that these osteolineage cells could be used for high-throughput screening technologies to develop new drugs to treat osteoporosis. In a recent advance in drug discovery for osteoporosis using treatment targeting sclerostin, it is indicated that the osteocyte must be one of the major targets [29].

In this study, we treated tissue-non-specific alkaline phosphatase (TNAP) positive osteolineage cells derived from hiPSCs, referred to as iPS-derived osteoprogenitors (iPSop), with 1,25(OH)_2_ vitamin D_3_. We compared iPSop cells with TNAP positive osteolineage cells derived from human mesenchymal stem cells (hMSCs), referred to as MSC-derived osteoprogenitors (MSCop). We found that osteocalcin (OCN), a late-phase marker for osteoblasts, was increased, and several osteocyte markers were detectable in iPSop cells but not in MSCop cells, after 1,25(OH)_2_ vitamin D_3_ treatment. Therefore, we suggest that iPSop cells can respond to osteogenic agents and may be a useful tool for drug assessment. Furthermore, this model could be used to develop new agents to promote osteogenic progression.

## 3. Material and methods

### 3.1. Cell culture and reagents

Human iPSCs (line 201B7; Riken Cell Bank, Tsukuba, Japan) were maintained with SNL76/7 feeder cells, clonally derived from a mouse fibroblast STO cell line transformed with neomycin resistance genes and murine LIF genes, in human ES medium (Dulbecco's modified Eagle's medium, nutrient mixture F-12 (DMEM/F-12, Invitrogen, Carlsbad, CA) with 20% knockout serum replacement (Invitrogen) supplemented with 1× non-essential amino acids solution (Chemicon, Temecula, CA), 2 mM l-glutamine (Chemicon), 1 mM 2-mercaptoethanol (Wako Pure Chemical Industries Ltd., Osaka, Japan), 1% penicillin/streptomycin (Invitrogen) and 5 ng ml^−1^ human fibroblast growth factor (FGF)-2 (ReproCELL Incorporated, Yokohama, Japan)). hMSCs were purchased Lonza (Basel, Switzerland), cultured in BulletKit MSC growth medium (Lonza) and used at passage 3 to 5. The 1,25(OH)_2_ vitamin D_3_ was purchased from Kyowa Hakko Kirin Co., Ltd. (Tokyo, Japan). Insulin-like growth factor (IGF)-1 and transforming growth factor (TGF)-β were purchased from Wako Pure Chemical Industries Ltd.

### 3.2. Embryoid body formation and *in vitro* osteogenic differentiation

The hiPSC colonies were dissociated with a cell scraper and transferred to low attachment Petri dishes to generate embryoid bodies (EBs). EBs were maintained in suspension in human ES medium without FGF-2 for 6 days. On day 6, the EBs were treated with 2 μM thiazovivin (Wako Pure Chemical Industries Ltd.) in human ES medium without FGF-2 for 1 h at 37°C and collected and dissociated in 0.5 mg ml^−1^ collagenase type IV (Wako Pure Chemical Industries Ltd.) for 20 min at 37°C, followed by treatment with 0.05% trypsin–EDTA (Invitrogen) for 5 min at 37°C. Cell suspensions were rinsed with α-MEM (Invitrogen, Carlsbad, CA) with 10% FBS, and cells were seeded onto cell culture dishes and cultured in osteoblast medium (OBM; α-MEM with 10% FBS, 50 μg ml^−1^ l-ascorbic acid (Wako Pure Chemical Industries Ltd.), 10 mM β-glycerophosphate (Wako Pure Chemical Industries Ltd.) and 10 nM dexamethasone (Wako Pure Chemical Industries Ltd.)). FGF-2, IGF-1 and TGF-β (FIT) were added on the following day (day 0) at a final concentration of 25 ng ml^−1^ FGF-2, 100 ng ml^−1^ IGF-1 and 1 ng ml^−1^ TGF-β. After culture in OBM with FIT for 0 (iPSop-day0), 4 (iPSop-day4), 10 (iPSop-day10) or 14 days (iPSop-day14), the cells were analysed and isolated by flow cytometry. In addition, the hMSCs were cultured in OBM with FIT and then isolated by flow cytometry on day 4 (MSCop-day4) or day 14 (MSCop-day14).

### 3.3. Flow cytometrical analysis and cell sorting

After washing with phosphate-buffered saline (PBS), the cells were treated with 0.05% trypsin–EDTA for 10 min at 37°C. The trypsinized cells were stained with phycoerythrin-conjugated anti-human alkaline phosphatase (ALP) antibody (R&D Systems, Minneapolis, MN) at a concentration of 10 μl/2 × 10^5^ cells for 45 min on ice. After staining, cells were washed three times with PBS, suspended in PBS containing 0.5% FBS, passed through a 40-μm mesh filter and maintained at 4°C until FACS sorting. We defined TNAP positive cells from hiPSCs and hMSCs as iPSop and MSCop, respectively. Dead cells were excluded by propidium iodide staining (2 μg ml^−1^) and forward scatter. Flow cytometrical analysis and cell sorting were performed on a FACS Aria (Becton-Dickinson, San Jose, CA).

### 3.4. Induction of osteoblast differentiation in osteoprogenitor-derived hiPSCs and hMSCs with 1,25(OH)_2_ vitamin D_3_

After culture in OBM with FIT for 14 days (iPSop-day14), the cells were isolated by flow cytometry, and 1,25(OH)_2_ vitamin D_3_ at a concentration of 10 or 50 nM was added on the following day (day 0). The medium was refreshed every 3 days, and the cells were analysed on day 6 and day 12. In addition, after culture in OBM with FIT for 4 days (iPSop-day4, MSCop-day4) or 14 days (iPSop-day14, MSCop-day14), iPSop and MSCop cells were isolated by flow cytometry and were treated with 50 nM 1,25(OH)_2_ vitamin D_3_. The medium was refreshed every 3 days, and the cells were analysed on day 6 and day 12.

### 3.5. mRNA expression

Total RNA was extracted using QIAzol reagent (Qiagen Inc., Valencia, CA) according to the manufacturer's instructions. cDNA was synthesized using a high-capacity cDNA reverse transcription kit (Applied Biosystems, Foster City, CA). Real-time RT-PCR (qRT-PCR) was performed using Premix Ex Taq reagent (Takara Bio Inc., Shiga, Japan) according to the manufacturer's instructions. Target genes included type I collagen (*COL1A1*), *TNAP*, runt-related transcription factor 2 (*RUNX2*), *OCN* and osterix (*OSX*). 18S rRNA was used as an internal control. All primers and probes are presented in table 1. Relative expression of genes of interest was estimated using the ΔΔ*Ct* method.

**Table 1.** Primers used for qRT-PCR.

gene symbol	GenBank accession no.	forward primer sequence	reverse primer sequence
*COL1A1*	NM_000088.3	5′-gggattccctggacctaaag-3′	5′-ggaaacctcgctctcca-3′
*TNAP*	NM_000478.3	5′-caaccctggggaggagac-3′	5′-gcattggtgttgtacgtcttg-3′
*RUNX2*	NM_001024630.2	5′-gtgcctaggcgcatttca-3′	5′-gctcttcttactgagagtggaagg-3′
*OCN*	NM_007541.2	5′-agactccggcgctacctt-3′	5′-ctcgtcacaagcagggttaag-3′
*OSX*	NM_152860.1	5′-catctgcctggctccttg-3′	5′-caggggactggagccata-3′
*18SrRNA*	M11188.1	5′-cggacaggattgacagattg-3′	5′-cgctccaccaactaagaacg-3′

RT-PCR was performed to examine some of the osteocyte-specific markers using ExTaq DNA polymerase (Takara Biotechnology, Shiga, Japan). Target genes included dentin matrix protein-1 (*DMP-1*), fibroblast growth factor-23 (*FGF-23*), matrix extracellular phosphoglycoprotein (*MEPE*) and podoplanin (*PDPN*). β-Actin was used as an internal control. Amplified PCR products were electrophoresed on 2% agarose gels. PCR primers used are listed in table 2.

**Table 2.** Primers used for RT-PCR.

gene symbol	GenBank accession no.	forward primer sequence	reverse primer sequence
*DMP-1*	NM_001079911	5′-caggagcacaggaaaaggag-3′	5′-ctggtggtatcttgggcact-3′
*FGF-23*	NM_020638.2	5′-tatttcgacccggagaactg-3′	5′-ggtatgggggtgttgaagtg-3′
*PHEX*	NM_000444.5	5′-aagaggaccctgggagaaaa-3′	5′-gggactgtgagcaccaattt-3′
*MEPE*	NM_001184694	5′-ccctttctgaagccagtgag-3′	5′-ttttcttcccccaggagttt-3′
*PDPN*	NM_001006624	5′-ccagcgaagaccgctataag-3′	5′-acgatgattgcaccaatgaa-3′
*β-actin*	NM_001101.3	5′-gggaaatcgtgcgtgacatta-3′	5′-ggcagtgatctccttctgcat-3′

### 3.6. Mineralization

The iPSop-day14 cells were treated with 1,25(OH)_2_ vitamin D_3_ for 2 weeks. The medium was removed, and the cells were rinsed in PBS and fixed in 4% paraformaldehyde for 5 min at room temperature. For mineralization, plates were treated with alizarin red solution for 5 min at room temperature. After 5 min, plates were rinsed in PBS and visually examined.

### 3.7. Statistical analysis

All data are expressed as the mean ± standard deviation (s.d.). When ANOVA indicated differences among the groups, multiple comparisons between each experimental group were performed using the Bonferroni test. Statistical significance was defined as *p* < 0.05.

## 4. Results

### 4.1. Induction of iPSop cells

**Figure 1.**
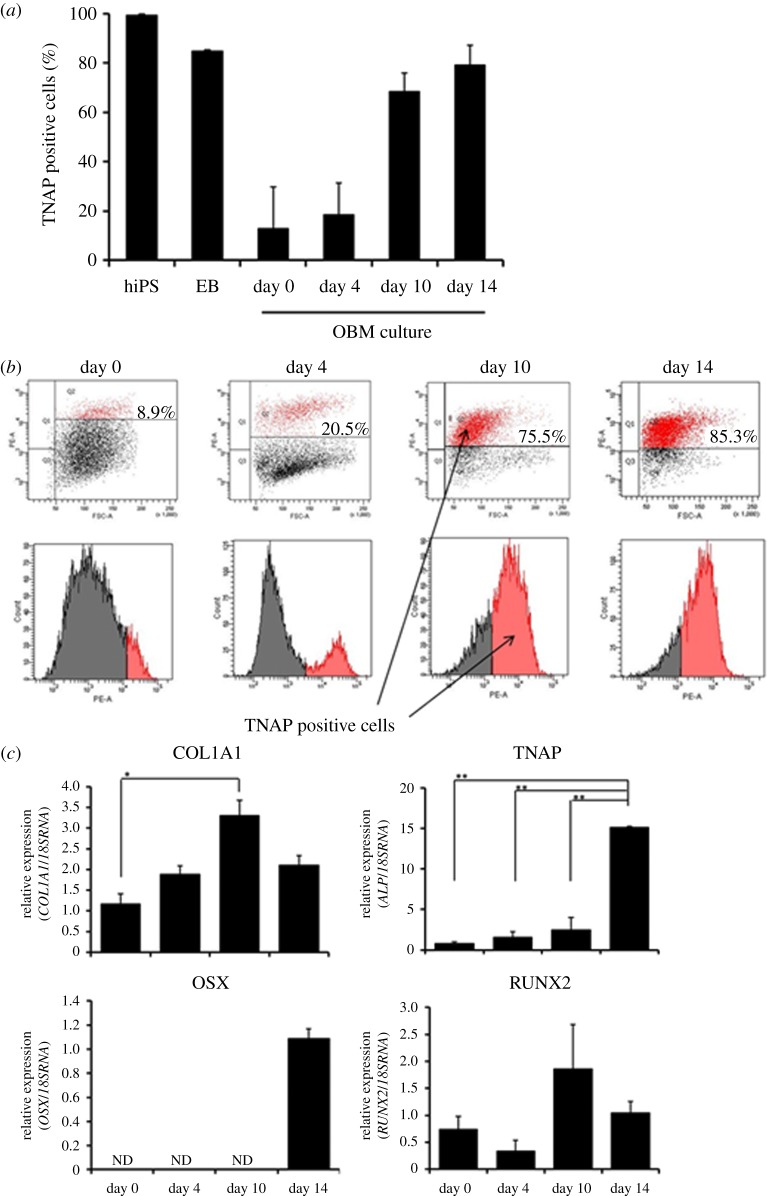
The expression of TNAP during hiPSC-to-osteogenic cell differentiation and the expression of osteoblast markers. (*a*) The frequency of TNAP positive cells in hiPSCs, EBs and single cells derived from EBs cultured in OBM with FGF-2/IGF-1/TGF-β (FIT). (*b*) Flow cytometrical analysis was performed on hiPSCs, EBs and single cells cultured in OBM with FIT for 0, 4, 10 and 14 days. (*c*) qRT-PCR analysis of *COL1A1, TNAP, OSX* and *RUNX2* was performed on iPSop-day0, 4, 10 and 14 cells. mRNA levels were normalized to those of 18S rRNA. Experiments were performed in triplicate. Values represent the mean ± s.d., *n* = 3. Bonferroni correction for multiple comparisons was applied. *p < 0.05, **p < 0.01.

As expected, the hiPSCs expressed a large amount of TNAP. We found that cells separated from EBs rapidly lost TNAP expression, but FIT treatment greatly increased TNAP expression (figure 1*a,b*). We isolated TNAP positive cells by FACS sorting and defined them as iPSop cells. Furthermore, we estimated osteoblast-specific markers in iPSop cells at each differentiation stage during culture in OBM. COL1A1 and TNAP expression increased over time. In iPSop-day10 cells, the expression of TNAP and RUNX2 was two times that in iPSop-day0 cells, but OSX expression was not detected. In iPSop-day14 cells, TNAP expression was further increased, RUNX2 expression was decreased and OSX expression sharply increased. Therefore, culture in OBM led to the differentiation of iPSop cells into osteoblasts over time (figure 1*c*). These results demonstrated that we could obtain osteoblasts at various stages of differentiation from iPSCs.

### 4.2. 1,25(OH)_2_ vitamin D_3_ promotes osteogenic differentiation of iPSop cells

**Figure 2.**
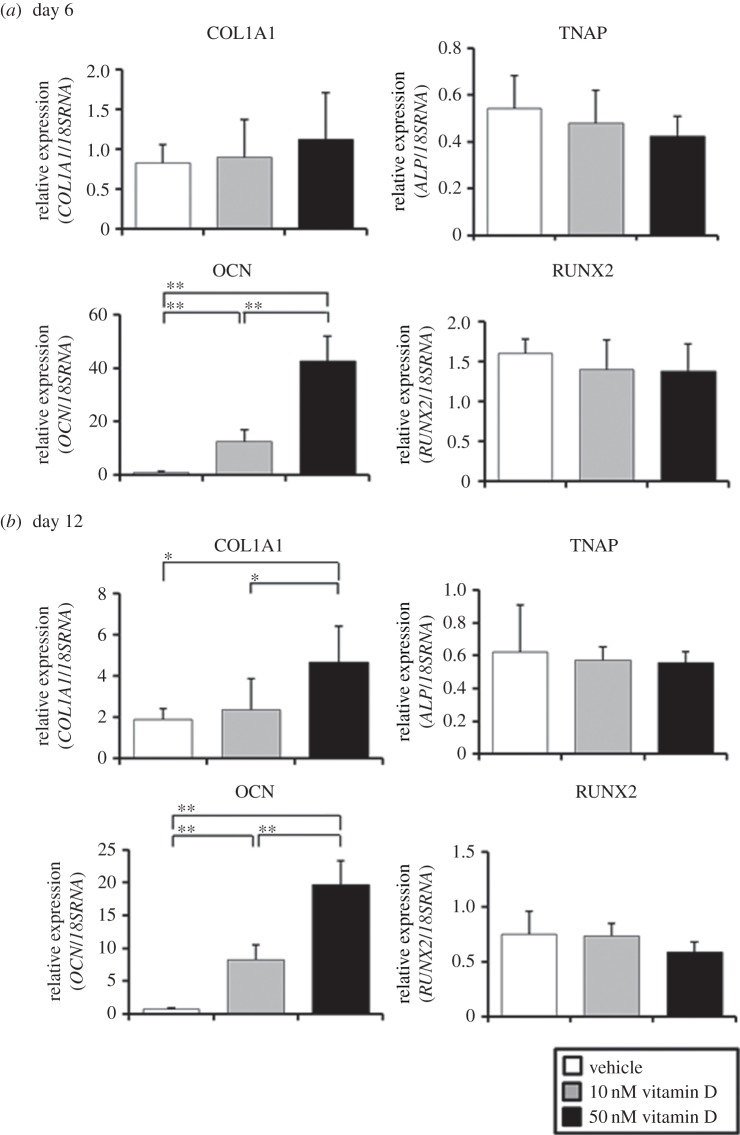
Expression of osteoblast markers after 1,25(OH)_2_ vitamin D_3_ treatment. (*a*) After isolation by flow cytometry, iPSop-day14 cells were treated with vehicle (control), 10 nM or 50 nM 1,25(OH)_2_ vitamin D_3_ for 6 days or (*b*) 12 days. Expression of *COL1A1, TNAP, OCN* and *RUNX2* was analysed with qRT-PCR, and mRNA levels were normalized to those of 18S rRNA. Experiments were performed in triplicate. Values represent the mean ± s.d., *n* = 4. Bonferroni correction for multiple comparisons was applied. *p < 0.05, **p < 0.01.

The treatment of iPSop-day14 cells with 1,25(OH)_2_ vitamin D_3_ for 6 days increased the expression of COL1A1 and OCN and decreased the expression of TNAP and RUNX2 (figure 2*a*). The increases or decreases in osteoblast markers occurred in a dose-dependent manner, and the longer the treatment, the more significant the change in osteoblast marker expression (figure 2*b*).

### 4.3. iPSop cells are more reactive to 1,25(OH)_2_ vitamin D_3_ than MSCop cells

**Figure 3.**
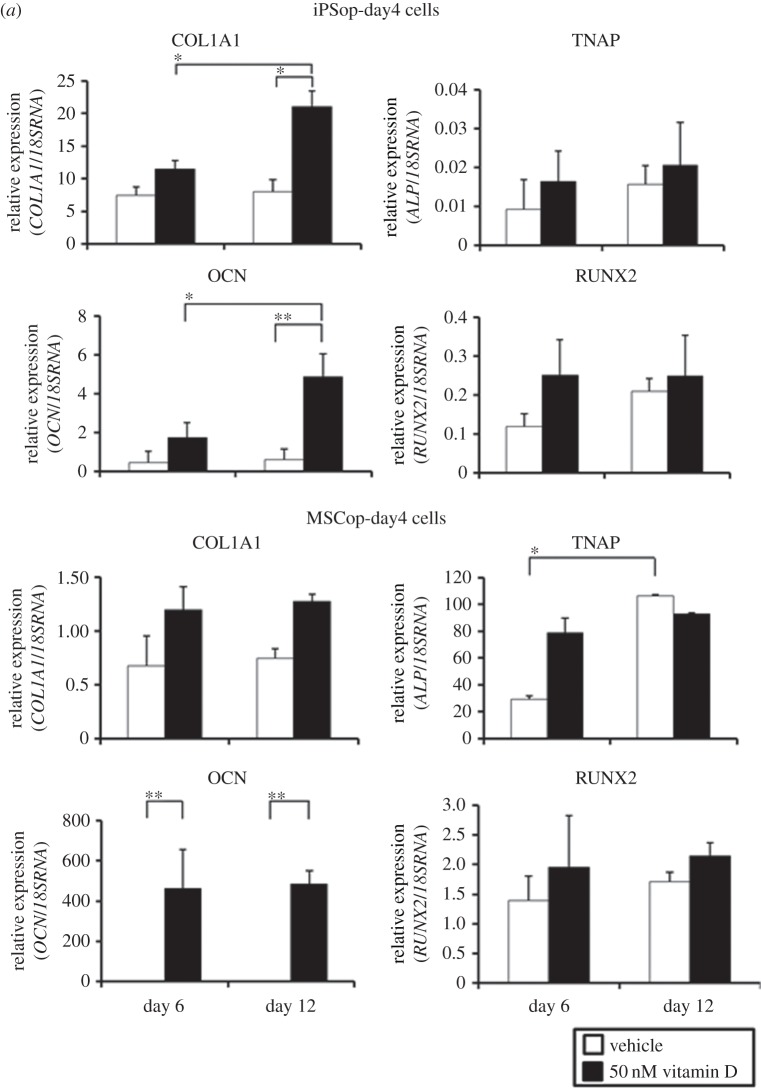

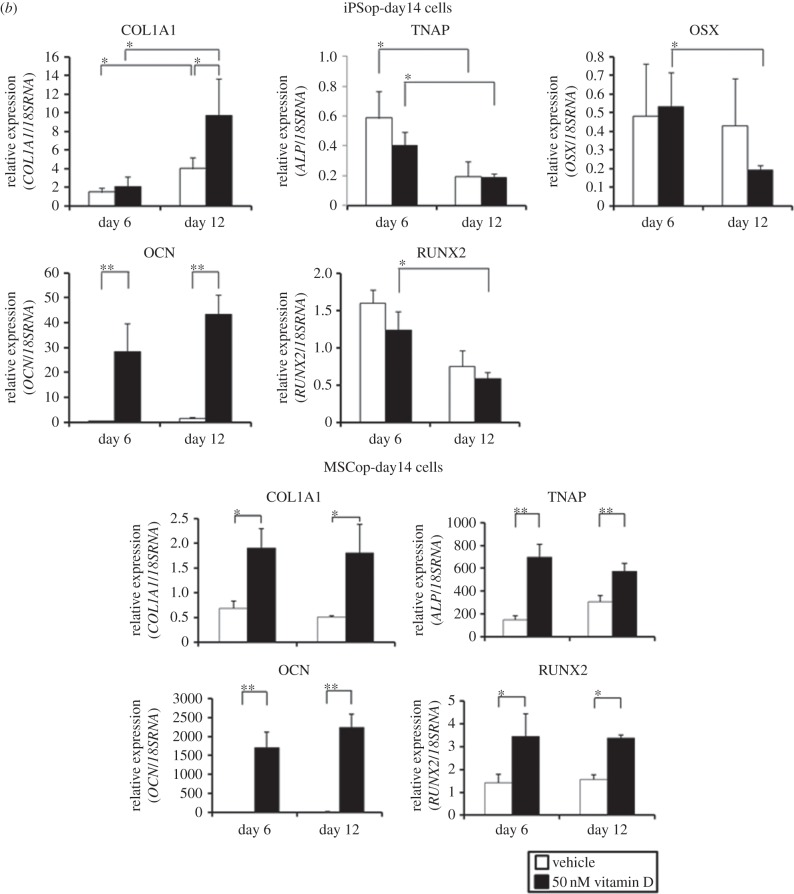
Expression of osteoblast markers in iPSop and MSCop cells at different differentiation stages after 1,25(OH)_2_ vitamin D_3_ treatment. iPSop and MSCop cells express various osteoblast markers. Comparison of expression of osteoblast marker genes between cells treated with vehicle and 1,25(OH)_2_ vitamin D_3_. (*a*) In iPSop- and MSCop-day4 cells. (*b*) In iPSop- and MSCop-day14 cells. These cells were treated with vehicle (white bar) or 50 nM 1,25(OH)_2_ vitamin D_3_ (black bar) for 6 or 12 days. Expressions of *COL1A1, TNAP, OCN, RUNX2* and *OSX* were analysed with qRT-PCR, and mRNA levels were normalized to those of 18S rRNA. Experiments were performed in triplicate. Values represent the mean ± s.d., *n* = 3. Bonferroni correction for multiple comparisons was applied. *p < 0.05, **p < 0.01.

Furthermore, we investigated the difference in response to 1,25(OH)_2_ vitamin D_3_ between iPSop cells and MSCop cells. We treated iPSop-day4 and MSCop-day4 cells, which were isolated by flow cytometry after culture in OBM with FIT for 4 days, with 1,25(OH)_2_ vitamin D_3_. Every osteoblast marker in MSCop-day4 cells increased but there was little difference between 6 and 12 days of 1,25(OH)_2_ vitamin D_3_ treatment (figure 3*a*). In iPSop-day4 cells, again every osteoblast marker increased with 1,25(OH)_2_ vitamin D_3_ treatment and marker expression continued to increase over time (figure 3*a*). In iPSop-day14 cells, expression of COL1A1 and OCN increased and expression of TNAP, RUNX2 and OSX decreased over time with 1,25(OH)_2_ vitamin D_3_ treatment (figure 3*b*). However, TNAP and RUNX2 expression in MSCop-day14 cells did not decrease with 1,25(OH)_2_ vitamin D_3_ treatment (figure 3*b*). These results suggest that iPSop cells were more reactive to 1,25(OH)_2_ vitamin D_3_ treatment than MSCop cells, and iPSop-day14 cells were advancing to a late phase of osteoblast differentiation.

### 4.4. iPSop cells expressed various osteocyte marker genes with 1,25(OH)_2_ vitamin D_3_ treatment

**Figure 4.**
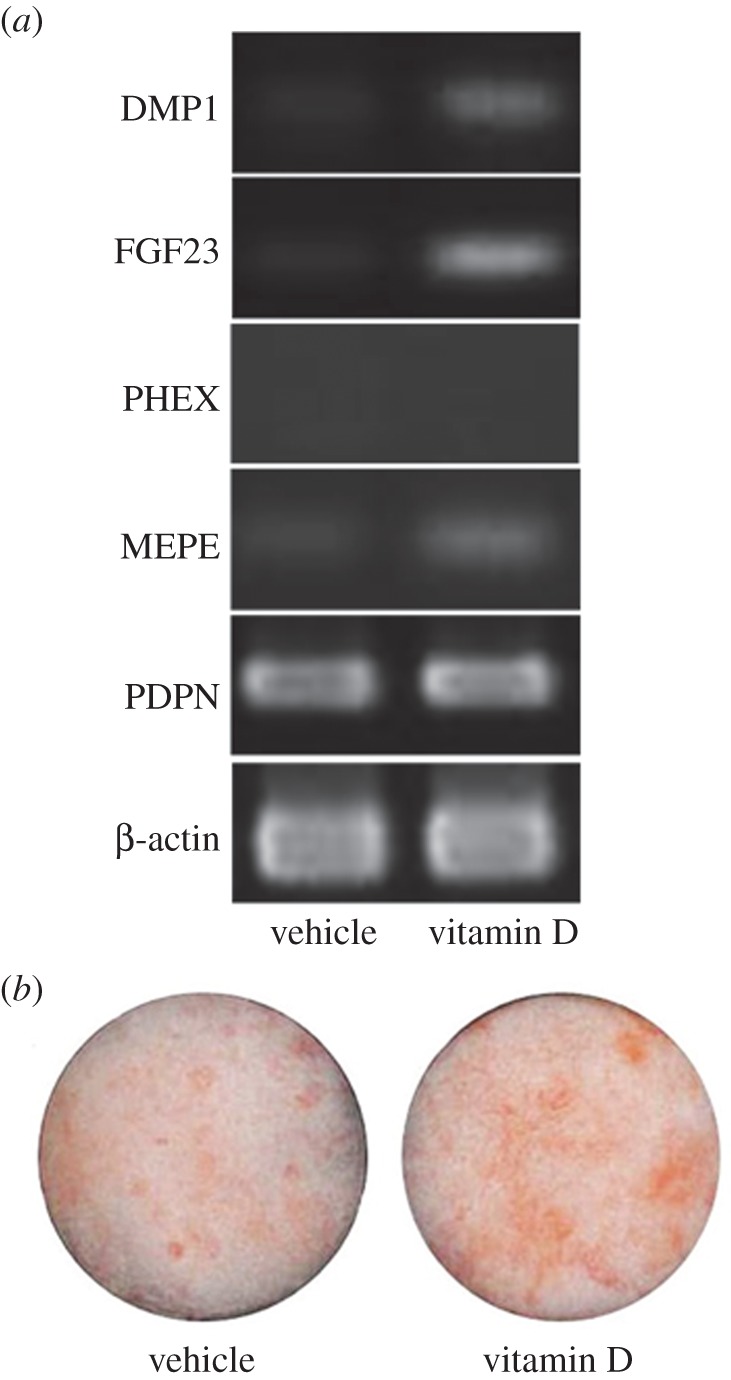
Expression of osteocyte-specific marker genes and the mineralization by alizarin red staining. (*a*) Expression of osteocyte-specific markers in iPSop-day14 cells treated with vehicle (vitamin D−) and 50 nM 1,25(OH)_2_ vitamin D_3_ (vitamin D+) for 14 days. The expression of dentin matrix protein-1 (*DMP-1*), fibroblast growth factor-23 (*FGF-23*), matrix extracellular phosphoglycoprotein (*MEPE*) and podoplanin (*PDPN*) were analysed by RT-PCR. *β-Actin* was used as an internal control. RT-PCR was performed using ExTaq DNA polymerase. Amplified PCR products were electrophoresed on 2% agarose gels. PCR primers are presented in table 2. (*b*) Mineralization in iPSop-day14 cells treated with vehicle (vitamin D−) and 50 nM 1,25(OH)_2_ vitamin D_3_ (vitamin D+) for 14 days with alizarin red staining.

Osteocyte-specific markers, such as DMP-1, FGF-23 and MEPE, were detected in iPSop-day14 cells treated with 1,25(OH)_2_ vitamin D_3_ for 14 days (figure 4*a*). In addition, 1,25(OH)_2_ vitamin D_3_ promoted mineralization in iPSop-day14 cells, when compared with vehicle alone (figure 4*b*). Therefore, these cells had proceeded to an early phase of osteocyte differentiation.

## 5. Discussion

Osteoporosis is a very common disease normally associated with ageing and caused by bone resorption overtaking bone formation. Dietary or supplemental calcium and activated vitamin D are required to reduce the risk of osteoporosis [15]. In clinical use, the intestinal calcium absorption effect of the prodrug alfacalcidol (1-hydroxyvitamin D_3_) becomes active at a daily dosage of 1.0 μg and then suppresses parathyroid hormone and inhibits bone resorption in adults. However, vitamin D has a narrow therapeutic window and bone resorption can occur at higher doses. Therefore, vitamin D may have both anabolic and catabolic effects on osteolineage cells. Although the mechanism underlying this biphasic effect is clinically important, to date, it is not well understood. The study of osteoremodelling by 1,25(OH)_2_ vitamin D_3_ has been limited by difficulty in obtaining osteocytes, which have a central role in that process.

We recently reported a new method to purify osteoprogenitors from hiPSCs. One of the unique advantages of our method is the ability to generate osteocyte-like cells. In this study, we used our system to evaluate the effects of 1,25(OH)_2_ vitamin D_3_ on osteoblast differentiation. First, we identified the optimal timing to obtain iPSop cells by sorting TNAP positive cells at different time points. Osteoblast marker gene expression differed over time. Cells liberated from hiPSC-derived EBs after 10 days of culture in OBM with FIT were sorted as TNAP positive cells and these cells showed increased expression of RUNX2. Four days later, after 14 days of culture in OBM with FIT, OSX expression was significantly increased (figure 1*c*). Therefore, it appears that cells liberated from hiPSC-derived EBs after 14 days of culture in OBM with FIT are differentiated into late-phase osteoblasts [34]. Next, we investigated the effects of 1,25(OH)_2_ vitamin D_3_ on these iPSop cells. We found that iPSop cells responded to 1,25(OH)_2_ vitamin D_3_ and the expression of osteoblast markers in these cells was affected in a dose-dependent manner. The effects of 1,25(OH)_2_ vitamin D_3_ on osteoblast marker gene expression suggested that iPSop-day4 cells were early-phase osteoblasts and iPSop-day14 cells were late-phase osteoblasts. Treatment with 1,25(OH)_2_ vitamin D_3_ increased all osteoblast markers in iPSop-day4 cells and MSCop-day4 cells. However, 1,25(OH)_2_ vitamin D_3_ treatment decreased the expression of some osteoblast markers (TNAP, RUNX2 and OSX) and increased OCN expression in iPSop-day14 cells but not in MSCop-day14 cells. Moreover, we observed an increase of mineralization in iPSop-day14 cells after 14 days of 1,25(OH)_2_ vitamin D_3_ treatment. We previously showed that it took 40 days for iPSop cells to express osteocyte markers [9]. The current observations clearly showed that 1,25(OH)_2_ vitamin D_3_ could be a potent accelerator of osteodifferentiation, particularly from late- to early-phase osteocytes. However, we did not detect an increase in sclerostin (an osteocyte-derived inhibitor of cultured osteoblasts) expression in this study. We suggest that high dose 1,25(OH)_2_ vitamin D_3_ treatment could induce sclerostin expression in newly formed osteocytes or existing osteocytes, which, in turn, could cause inhibition of Wnt signalling and accelerate the resorption of bone tissue.

Another important point is that this method could be used for research into new drugs for osteoporosis. Human-cell-based *in vitro* assay systems are a basic requisite for drug discovery and iPSCs reprogrammed from somatic cells can provide an opportunity to establish these systems [3,4]. Recent research has revealed that osteocytes are part of the command centre for osteoremodelling, and one reason is that sclerostin is exclusively secreted from osteocytes [35]. A neutralizing antibody to sclerostin has been shown to be effective in initial preclinical and early clinical trials. Postmenopausal women treated with a neutralizing antibody to sclerostin showed an increase in markers of bone formation [29]. Therefore, agents regulating osteocyte functions such as sclerostin secretion could be effective in the treatment of osteoporosis. However, screening methods using animal models may not reflect the human situation and some compounds found to be successful in cellular or animal models have failed to show effectiveness in clinical trials [35,36]. It has been postulated that drug screening using iPSCs could identify compounds that increase differentiation and promote maturation in target cells [36]. To date, there are few examples of the use of high-throughput screening methods to assess differentiation. We believe that the iPSop cells generated using our method could be a valuable cell source for high-throughput screening to research new drugs for a range of diseases, including osteoporosis.

## 6. Conclusion

Treatment with 1,25(OH)_2_ vitamin D_3_ could promote osteogenic differentiation in iPSop cells and could accelerate the expression of osteocyte marker genes. This is the first report showing that 1,25(OH)_2_ vitamin D_3_ can promote osteocyte generation. Because iPSCs expand into a large number of cells, this method could be used for high-throughput screening of new drugs to treat osteoporosis. We hope that our method will provide us a new platform for research into the treatment of osteoporosis and other bone diseases.
